# Prospective evaluation of mpMRI-derived nomograms for detecting prostate cancer in PI-RADS v2.1 upgraded and non-upgraded lesions

**DOI:** 10.3389/fonc.2025.1510049

**Published:** 2025-06-04

**Authors:** Ying Yi, Hang Wang, Dongliang Cheng, Zhifeng Xu, Xianhai Zhang, Chun Luo, Hai Zhao

**Affiliations:** Department of Radiology, First People’s Hospital of Foshan, Foshan, China

**Keywords:** prostate cancer, multiparametric magnetic resonance imaging, nomogram, predictive model, synthetic MRI, PI-RADS v2.1

## Abstract

**Background:**

Limited data exist on the performance of Prostate Imaging Reporting and Data System (PI-RADS) v2.1 upgraded and non-upgraded lesions, both alone and in combined with multiparametric MRI (mpMRI) features, for prostate cancer detection.

**Objective:**

To evaluate the rates of prostate cancer (PCa) and clinically significant prostate cancer(csPCa) rates in PI-RADS v2.1 upgraded and non-upgraded lesions, and to identify mpMRI features that improve detection accuracy.

**Methods:**

This study included men who underwent mpMRI and ultrasound-guided (US-guided) biopsy from March 2023 to April 2024. MRI scans were prospectively evaluated according to PI-RADS v2.1. MpMRI features were extracted from lesion contours, including three-dimensional maximum diameter, lesion volume, sphericity, surface-to-volume ratio (SVR), T_2_-weighted imaging signal intensity(T_2_WI SI), diffusion-weighted imaging(DWI) SI, T_1_, T_2_, proton density (PD), apparent diffusion coefficient (ADC), and dynamic contrast-enhanced (DCE) MRI-derived time intensity curve (TIC). Univariable and multivariable logistic regression analyses were performed to identify features associated with PCa and csPCa in different prostate zones (transition zone and peripheral zone).

**Results:**

A total of 94 patients(mean age, 65.7 years) with 234 lesions were included. Significant differences were observed between upgraded and non-upgraded PI-RADS 4 lesions(*p* < 0.05) in the peripheral zone (PZ), whereas no significant differences were found in the transition zone (TZ). Risk factors for csPCa in the TZ included lesion diameter, TIC type III, capsule, T_1_ and PD values. For csPCa in the PZ, T1, SVR, DWI SI, and ADC values were identified as important risk factors. ROC analysis demonstrated high diagnostic accuracy for csPCa detection, with AUCs of 0.93 (TZ) and 0.96 (PZ).

**Conclusion:**

PI-RADS v2.1 upgrading rules improve cancer detection in the TZ, but upgrading PI-RADS category 3 lesions in the PZ may lead to unnecessary biopsies. MpMRI-based nomograms enhance predictive accuracy for both PCa and csPCa.

## Introduction

Prostate cancer (PCa) remains one of the most rapidly increasing malignancies globally, with significant implications for public health (1). Multiparametric magnetic resonance imaging (mpMRI) has emerged as a cornerstone in guiding prostate biopsy decisions, enabling targeted sampling of suspicious lesions (2, 3). Despite its clinical utility, the Prostate Imaging Reporting and Data System (PI-RADS) v2.1 has certain limitations, including moderate interobserver variability and suboptimal accuracy in detecting clinically significant prostate cancer (csPCa)—particularly when applying its upgrading rules. These rules allow dominant lesion scores to be elevated to higher final categories, potentially influencing biopsy thresholds (4).

Recent advances in quantitative MRI biomarkers—such as synthetic MRI (SynMRI), multidimensional morphological features (e.g., lesion volume, sphericity), apparent diffusion coefficient (ADC) value, and dynamic contrast-enhanced MRI (DCE-MRI)-derived time-intensity curve (TIC)—have shown promise in refining diagnostic precision. SynMRI, an innovative technique generating quantitative T1, T2 and proton density (PD) maps, complements conventional mpMRI by enhancing tissue characterization (5, 6). Studies integrating these parameters have demonstrated improved discrimination of tumor aggressiveness and pathological staging (7, 8), suggesting their potential to mitigate PI-RADS limitations.

Building on this evidence, this study aims to: (i) compare PCa and csPCa detection rates between PI-RADS v2.1 upgraded and non-upgraded lesions, and (ii) identify predictive mpMRI features across prostate zones—the transition zone (TZ) and peripheral zone (PZ).

## Methods

### Participants population

**Figure 1:**
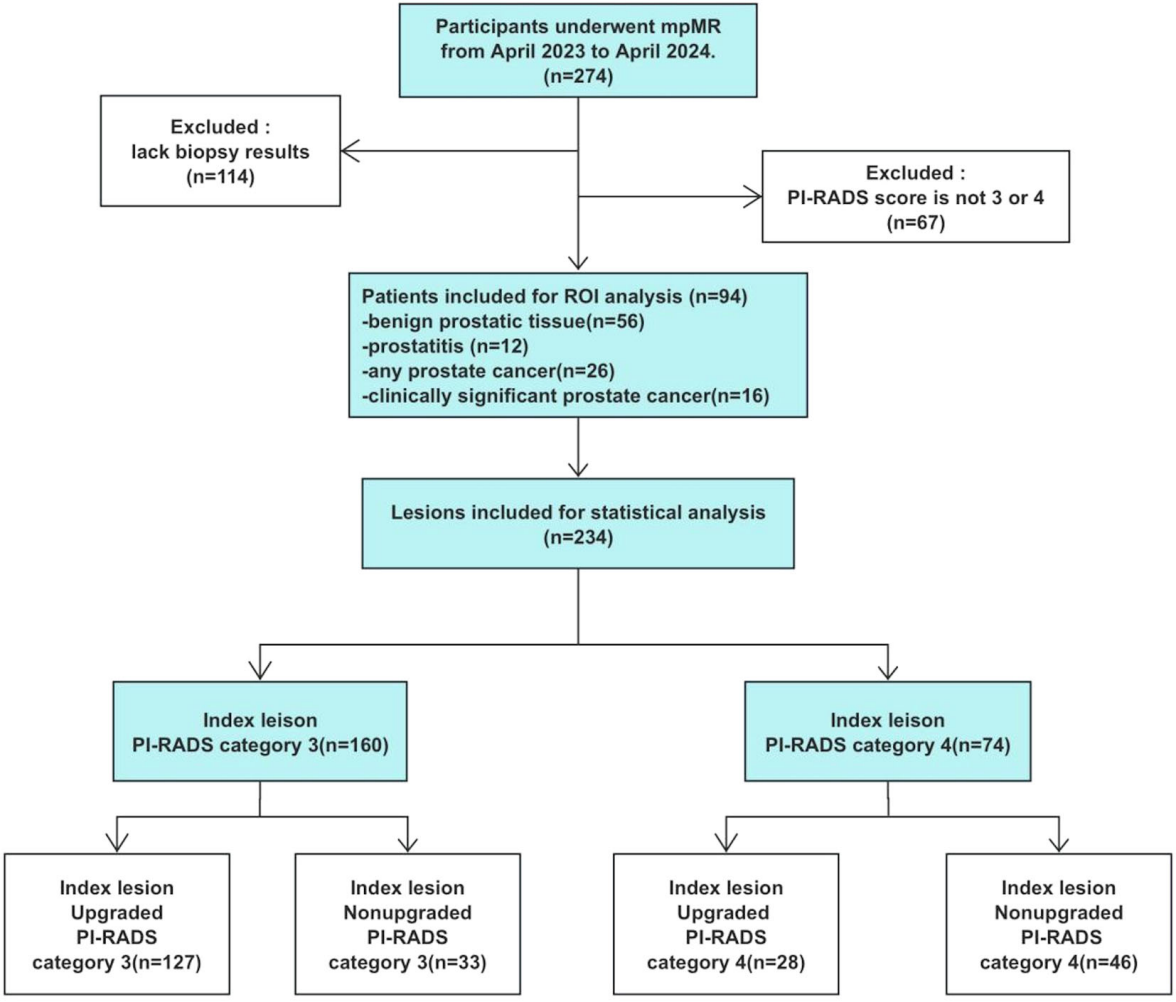
Flowchart shows study inclusion and exclusion. mpMRI, multiparametric MRI; PI-RADS, Prostate Imaging Reporting and Data System; ROI, region of interest.

**Figure 2:**
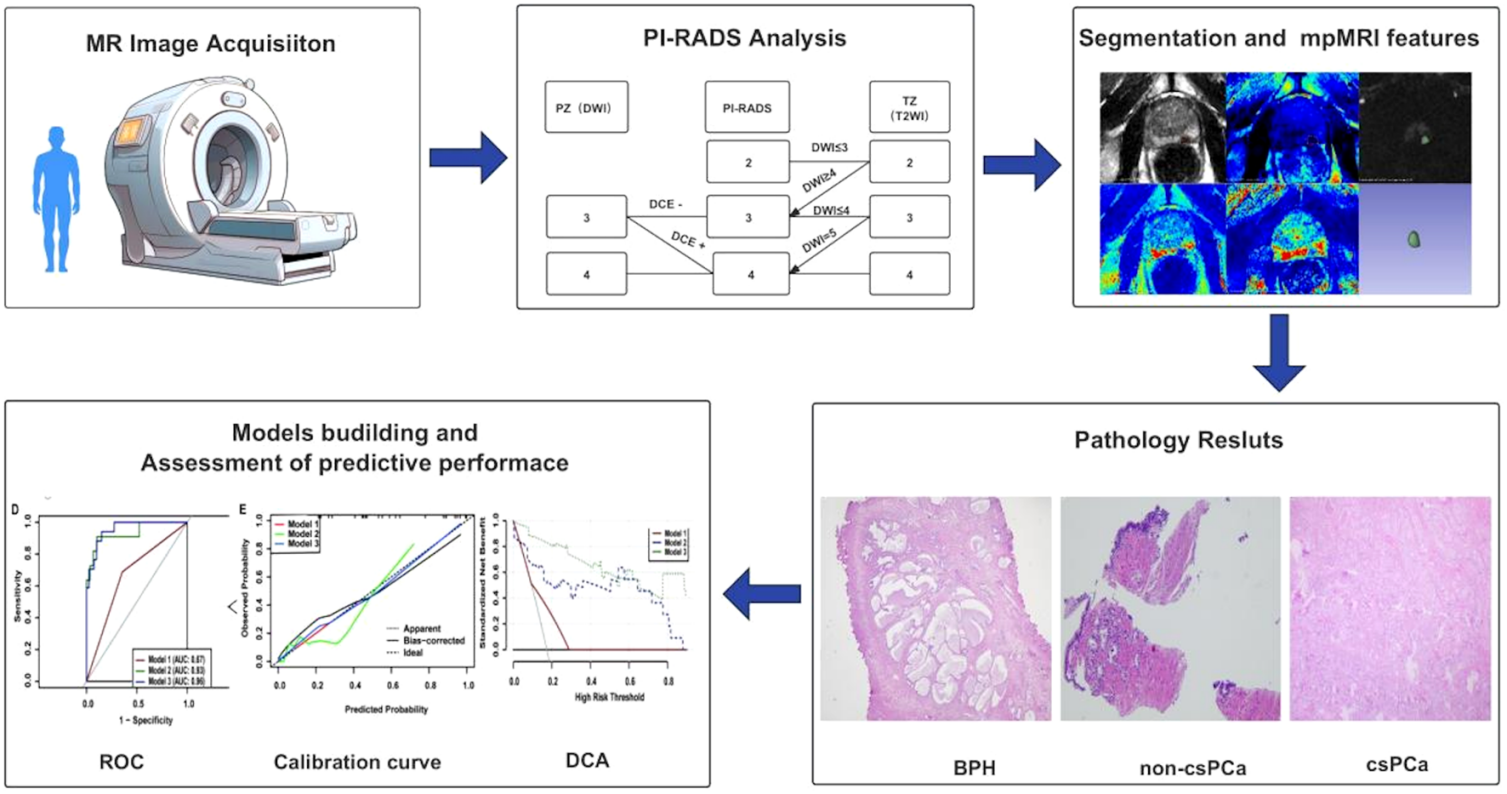
Workflow for this study. mpMRI, multiparametric MRI; PI-RADS, Prostate Imaging Reporting and Data System.

This prospective study was approved by our institutional review board, and written informed consent was obtained from all participants. Consecutive participants with elevated prostate-specific antigen (PSA) levels who underwent multiparametric MRI (mpMRI) at an academic medical center from April 2023 to April 2024 were enrolled. Participants without biopsy results or those not categorized as PI-RADS 3 or 4 were excluded. All treatment-naive participants meeting the inclusion criteria were deemed eligible and included in the final study cohort (**Figures 1**, **2**). All participants underwent transperineal (TP) ultrasound-guided systematic prostate biopsy in the urology department. The systematic biopsy was conducted following the Ginsburg protocol, with a minimum of 10 cores obtained (9). CsPCa was defined as International Society of Urological Pathology (ISUP) grade groups ≥2 (range, 1-5), a targeted prostate mapping (TPM) cancer core length (CCL) ≥ 6 mm, or extracapsular extension (10). Urinary tract infection was diagnosed based on white blood cell (WBC) count > 10 per high-power field in routine urinalysis (11).

### Image acquisition

All MR examinations were performed using a 3.0T scanner (SIGNA Architect; GE Healthcare) with a 32-channel cardiac phased-array coil. T_2_WI, DWI and Synthetic MRI sequences were acquired before injecting Gadopentetate dimeglumine (Guang Zhou CONSUN Pharmaceutical CO., LTD). The dynamic contrast-enhanced(DCE) MRI sequence was performed using a 3D T_1_-weighted gradient-recalled echo technique, with a temporal resolution of 12 seconds for the first 3 phases, followed by the subsequent 17 phases. Details image acquisition parameters are provided in **Supplementary Table 1**.

### Subjective analysis

Prospective mpMRI examinations were interpreted by two radiologists (X.H.Z and Y.Y, with 12 and 3 years of experience in prostate MRI interpretation, respectively), based on PI-RADS version 2.1 independently and blinded to the final histopathology results. The number of lesions and the PI-RADS score for each lesion were recorded separately. If the two radiologists assigned different PI-RADS scores to the same lesion, the final score was determined by a third radiologist (H.Z., with 22 years of experience in prostate MRI interpretation). According to PI-RADS version 2.1, a lesion can be upgraded to a higher category under the following conditions: (a) a PZ lesion with a DWI (dominant sequence for PZ) score of 3 shows positive dynamic contrast enhancement; (b) a TZ lesion with a T2WI score of 3 has a DWI score of 5; or (c) a TZ lesion with a T2WI score of 2 has a DWI score of 4 or higher. Homogeneous signal intensity and capsule integrity were assessed on T2WI, while diverse lesions were evaluated on DWI, specifically for TZ lesions. Diverse lesions are defined as discrete and distinct from the background, visible only on high b value DWI(b ≥ 1400 s/mm^2^) (12).

### Shape features and quantitative features

**Shape features**—Following subjective analysis, each radiologist independently segmented the lesions for quantitative shape analysis. Axial T2WI and DWI image were exported in DICOM format from PACS to an independent workstation for lesion segmentation using 3D Slicer version 5.6.2 (https://www.slicer.org). Radiologists manually delineated the outer contours of each nodule on successive slices, saving these contours as volumes of interest (VOIs). Shape features included lesion volumes, relative lesion volume (individual lesion volume divided by prostate volume), three-dimensional diameter (the diameter of a sphere encompassing the entire segment), surface-to-volume ratio (SVR), roundness, elongation, and flatness. Roundness (range: 0–1) was calculated as the ratio of the surface area of a sphere (derived from the 3D diameter) to the actual surface area of the lesion. Lower SVR values and higher roundness values indicate a more spherical lesion shape. It should be noted that SVR is volume-dependent; when comparing a small and a large lesion with identical shapes, the SVR tends to be larger in small lesion (13). Other shape features, such as elongation and flatness, range from 0 to 1. Lesions that are elongated and flat are characterized by lower elongation and flatness values (14).

**Quantitative features**—Prostate volume (PV) was computed using the formula: anteroposterior diameter × vertical diameter × transverse diameter × 0.52 (12). PSA density(PSAD) was determined by dividing the PSA by the PV (15). Regions of interest (ROIs) was manually delineated on T2WI or DWI, depending on the lesion’s location. These ROIs were cloned to other mpMRI sequences to acquire ADC, DWI signal intensity(SI), T2WI SI, and TIC. ADC maps were automatically derived from the DWI images(b=0, 1000) (16). The mean T2WI SI of the lesion was normalized by the internal obturator muscle (hereafter, relative T2WI SI). The DWI signal of the lesion was normalized by the whole prostate parenchyma (hereafter, relative DWI SI). Time-intensity curves were classified into three subtypes: type I (wash-in), type II (wash-out stability), and type III (wash-out) (17). For analysis, type I and II were grouped together, while type III was analyzed separately. Quantitative parametric maps (T1, T2, and PD) were generated from MDME raw data using post-processing software (SyMRI 8.0; Synthetic MR, Linkoping, Sweden). According to a previous study (18), we adopted T1 ≤1151.27ms, T2 ≤84.42ms and PD ≤74.2pu as subgroup references for TZ and T1 ≤1248.35ms, T2 ≤90.25ms and PD ≤80.19pu for PZ.

### Statistical analysis

Descriptive statistics were presented as mean ± standard deviation(SD) for normally distributed variables and median with interquartile range (IQR) for non-normally distributed variables. The Chi-square (χ²) test, modified χ2 test and Fisher test were employed to compare PCa and csPCa detection rates between PI-RADS upgraded and non-upgraded lesions, with 95% confidence intervals (CIs) estimated for lesion-based cancer detection rates (19). Variables with *p <* 0.05 in univariate analysis were subsequently included in multivariate logistic regression models to identify independent predictors of cancer detection (13). A backward stepwise procedure based on the minimum Akaike information criterion(AIC) was applied to the final multivariable model (20). Nomograms were constructed based on the results of these regressions.

The performance of nomograms was evaluated using odds ratios (ORs), 95% confidence intervals (CIs), and *p* values. Discriminative ability was assessed using the area under the receiver operating characteristic curve (AUC). Calibration curves were plotted to compare predicted probabilities with observed outcomes; a calibration curve closely following the 45-degree line indicated good agreement. Decision curve analysis (DCA) was applied to evaluate the clinical net benefit of the models. All statistical analyses were performed using R (version 4.4.0) and Zstats 1.0 (www.zstats.net). All tests were two-sided and *p <*0.05 was considered statistically significant.

## Results

### Participant characteristics

Among 94 patients, 56 (59.6%) were diagnosed with benign prostatic tissue, 12(12.8%) with prostatitis, 26 (27.7%)with PCa, of which 17 (18.1%) were classified as csPCa. The mean age of participants was 65.7 ± 8.6 years, with a median serum PSA level of 11.0 ng/mL (IQR, 7.6-15.2) and a median PV level of 58.9 mL (IQR, 39.6-78.0). The median PSAD was 0.22 ng/ml/cm³(IQR, 0.13-0.30). Among all patients, 55 (58.5%) had a highest PI-RADS score of 3, while 39 (41.5%) were categorized as PI-RADS 4. Most participants (85%) were biopsy-naive, with only 14 (15%) having a history of prior negative biopsies. The median interval between MRI and biopsy was 7 days (IQR, 4–11 days). Urinary tract infection was diagnosed in 32 (34%) participants, and 62 (66%) had negative urinalysis results. Fifty (53%) participants had diverse lesions, while 44 (47%) did not.

### Cancer detection rates of PI-RADS version 2.1 upgraded versus non-upgraded categories

**Figure 3:**
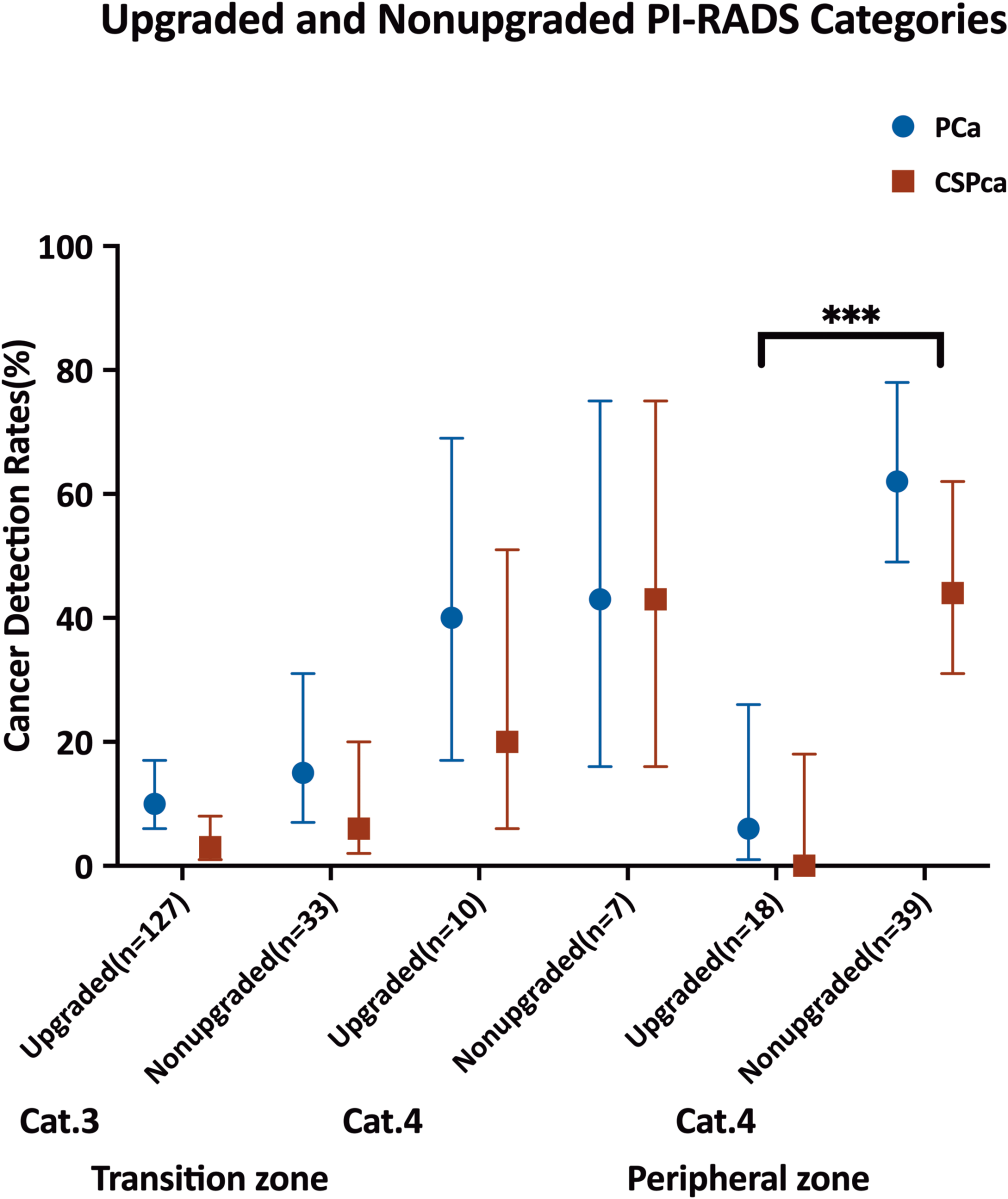
Dot plot with 95% CIs show lesion-based cancer detection rates for all prostate cancer (PCa) and clinically significant PCa (csPCa) in Prostate Imaging Reporting and Data System (PI-RADS) non-upgraded and upgraded categories. Braces indicate statistically significant differences between consecutive PI-RADS categories. *** indicates *p ≤*.001 for both PCa and csPCa.

Among the lesions evaluated, 160 were classified as PI-RADS 3 lesions and 74 as PI-RADS 4 lesions. Specifically, 79% (127 out of 160) of PI-RADS 3 lesions and 38% (28 out of 74) of PI-RADS 4 lesions were upgraded according to PI-RADS version 2.1 guidelines.Significant differences in detection rates were observed only in the PZ, where upgraded lesions had notably higher detection rates of PCa (62% [24 of 39] vs. 6% [1 of 18], *p* < 0.001) and csPCa (44% [17 of 39] vs. 0% [0 of 18], *p* = 0.001) compared to non-upgraded lesions. Conversely, in the TZ, detection rates of PCa (10% [13 out of 127] vs. 15% [5 out of 33], *p* =0.426) and csPCa (3% [4 out of 127] vs. 6% [2 out of 33], *p* = 0.787) were similar between upgraded and non-upgraded PI-RADS 3 lesions. Similarly, among PI-RADS 4 lesions in the TZ, there were no significant differences in the detection of PCa (40% [4 out of 10] vs. 43% [3 out of 7], *p* = 1.000) or csPCa (20% [2 out of 10] vs. 43% [3 out of 7], *p* = 0.593) between upgraded and non-upgraded lesions among PI-RADS 4 lesions in the TZ (**Table 1**; **Figure 3**).

**Table 1:** Non-upgraded and upgraded PI-RADS categories.

Zone and Category	subgroups	No. of lesions	No. of PCa	PCa Rate (%)	95% CI	*p* value	No. of csPCa	csPCa Rate (%)	95% CI	*p* value
TZ-Cat.3	upgraded	127	13	10	6-17	0.426	4	3	1-8	0.787
	non-upgraded	33	5	15	7-31		2	6	2-20	
TZ-Cat.4	upgraded	10	4	40	17-69	1.000	2	20	6-51	0.593
	non-upgraded	7	3	43	16-75		3	43	16-75	
PZ-Cat.4	upgraded	18	1	6	1-26	**<.001**	0	0	0-18	**0.001**
	non-upgraded	39	24	62	49-78		17	44	31-62	

PCa, prostate cancer; csPCa, clinically significant prostate cancer; PZ, peripheral zone; TZ, transition zone. Values in bold indicate statistical significance (*p* < 0.05).

### Lesion-specific mpMRI features for TZ

**Figure 4:**
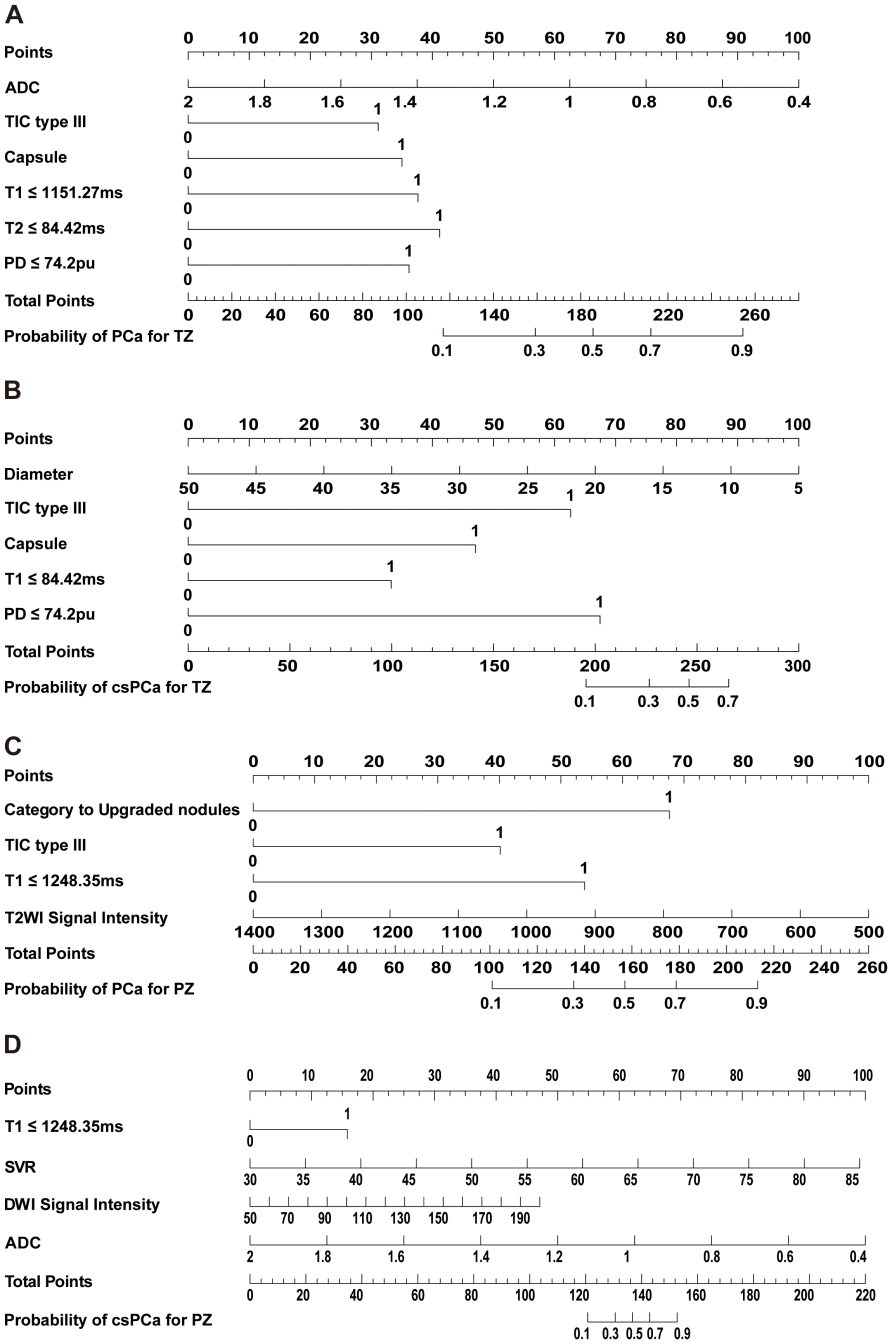
Nomograms to predict the probability of prostate cancer(PCa) and clinically significant prostate cancer (csPCa) for transitional zone(TZ) and peripheral zone (PZ). **(A)** Nomogram to predict the probability of PCa for TZ. **(B)** Nomogram to predict the probability of clinically significant prostate cancer for TZ. **(C)** Nomogram to predict the probability of PCa for PZ. **(D)** Nomogram to predict the probability of clinically significant prostate cancer for PZ. TIC, time intensity curve.

**Figure 5:**
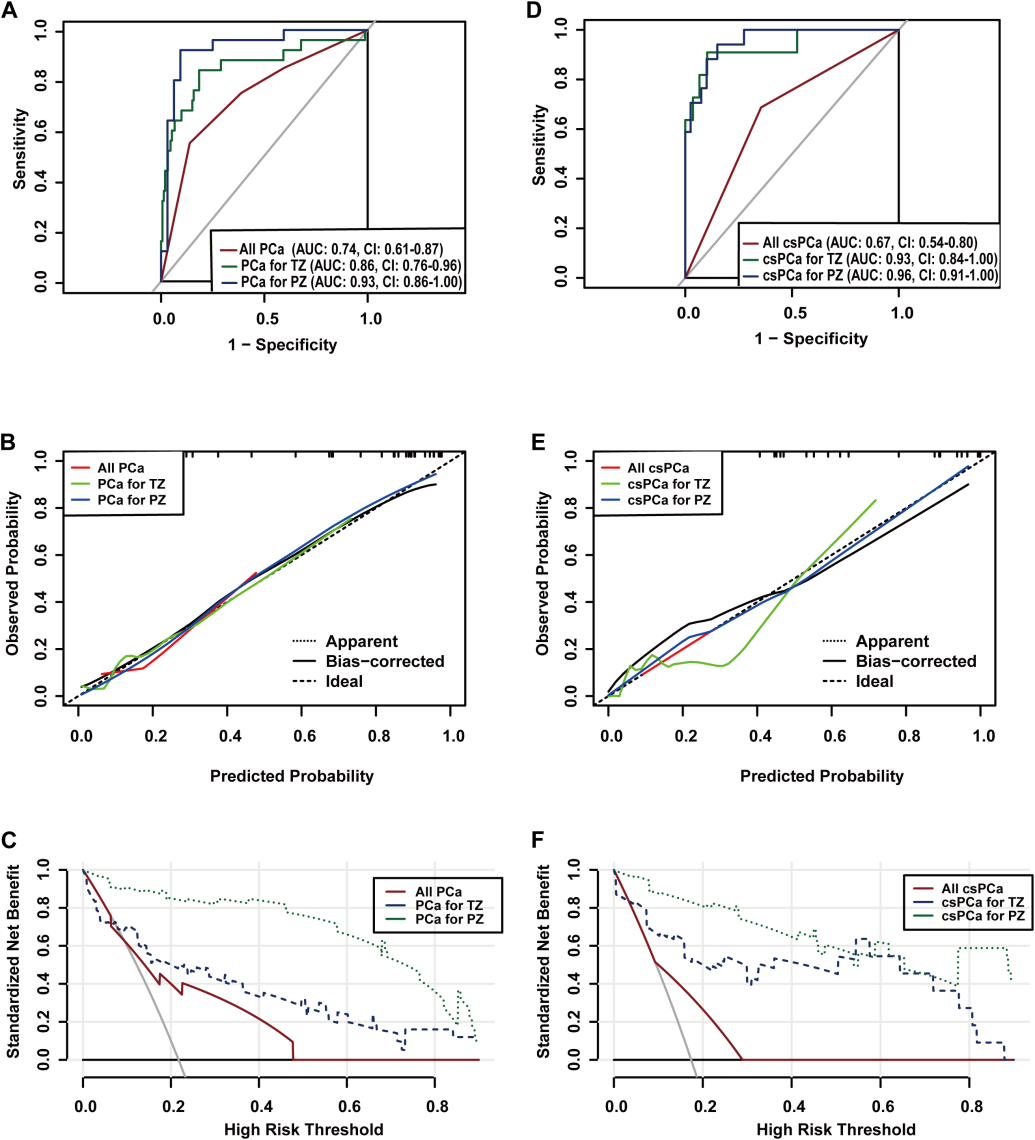
The area under the receiver operating characteristic curve(AUC) **(A)**, calibration curve **(B)** and decision curve **(C)** of the prediction models for PCa. Model 1 for all PCa detection based on Diverse lesions and Highest PIRADS category, Model 2 for Transition zone PCa detection based on Time intensity curve(TIC) type III(wash-out), Capsule, T1, T2, PD, and ADC, and Model 3 for Peripheral zone PCa detection based on Category to Upgraded lesions, TIC type III, T1, and T2WI Signal Intensity. And the AUC **(D)**, calibration curve **(E)** and decision curve **(F)** of the prediction models for csPCa. Model 1 for all csPCa detection based on Highest PIRADS category, Model 2 for Transition zone csPCa detection based on Diameter, TIC type III, Capsule, T1, and PD, Model 3 for Peripheral zone csPCa detection based on SVR, T1, DW signal intensity, and ADC.

In the TZ group, 14.1% (25/177) of patients were diagnosed with PCa, with only 6.2% (11/177) diagnosed with csPCa. Multivariate analysis identified TIC type III (wash-out) (OR= 2.70, [95% CI: 0.92 ~ 7.95], *p* =0.071), absence of capsule(OR= 3.06, [95% CI: 1.01 ~ 9.37], *p* =0.050), T1 ≤1151.27ms(OR= 3.33, [95% CI: 0.95 ~ 11.66], *p* =0.060), T2 ≤84.42ms(OR= 3.73, [95% CI: 1.24 ~ 11.22], *p* =0.019) and PD ≤74.2pu(OR= 3.18, [95% CI: 1.05 ~ 9.60], *p* =0.040)and lower ADC (OR= 0.14, [95% CI: 0.02 ~ 1.22], *p* =0.074). For csPCa detection, TIC type III (wash-out) (OR=15.18, [95% CI: 2.32 ~ 99.21], *p* =0.005), absence of capsule(OR=7.71, [95% CI: 1.29 ~ 46.21], *p* =0.025), T1 ≤1151.27ms(OR=4.23, [95% CI: 0.78 ~ 22.85], *p* =0.093), PD ≤74.2pu (OR=18.69, [95% CI: 1.57 ~ 221.96], *p* =0.020) and diameter (OR=0.91, [95% CI: 0.81 ~ 1.01], *p* =0.087) were risk factors (**Table 2**). Nomograms were developed based on the final logistic regression models to assess the risk of PCa and csPCa (**Figures 4A, B**), achieving AUCs of 0.86 (95% CI: 0.76 ~ 0.96) and 0.93 (95% CI: 0.84 ~ 1.00), respectively. DCA demonstrated superior benefit across most threshold ranges. Calibration plots of predicted probabilities for PCa and csPCa (**Figure 5**).

**Table 2:** Lesion-level univariable and multivariable analyses for all prostate cancer and clinically significant prostate cancer in TZ.

Variable	Prostate Cancer		Variable	Clinically Significant Prostate Cancer	
	Multivariable Analysis			Multivariable Analysis	
	OR	*p* value		OR	*p* value
TIC			TIC		
Type I and II	Reference		Type I and II	Reference	
Type III	2.70 (0.92 ~ 7.95)	0.071	Type III	15.18 (2.32 ~ 99.21)	**0.005**
Capsule			Capsule		
Yes	Reference		Yes	Reference	
No	3.06 (1.01 ~ 9.37)	**0.050**	No	7.71 (1.29 ~ 46.11)	**0.025**
T1			T1		
>1151.27ms	Reference		>1151.27ms	Reference	
≤1151.27ms	3.33 (0.95 ~ 11.66)	0.060	≤1151.27ms	4.23 (0.78 ~ 22.85)	0.093
T2			PD		
>84.42ms	Reference		>74.2ms	Reference	
≤84.42ms	3.73 (1.24 ~ 11.22)	**0.019**	≤74.2ms	18.69 (1.57 ~ 221.96)	**0.020**
PD			Diameter	0.91 (0.81 ~ 1.01)	0.087
>74.2pu	Reference				
≤74.2pu	3.18 (1.05 ~ 9.60)	**0.040**			
ADC	0.14 (0.02 ~ 1.22)	0.074			

Data in parentheses are 95% CIs. OR, odds ratio; TZ, transitional zone; TIC, time intensity curve; type I(wash-in), type II (wash-out stability), and type III(wash-out). Values in bold indicate statistical significance (*p* < 0.05).

### Lesion-specific mpMRI features for PZ

Among 57 PZ lesions, the detection rates for PCa and csPCa were 43.9% and 29.8%, respectively. non-upgraded lesions(OR=3.33, [95% CI: 0.95 ~ 11.66], *p* = 0.045), *p* = 0.045), TIC type III (wash-out) (OR=4.82, [95% CI: 0.78 ~ 29.64], *p* = 0.090), T1 ≤1248.35ms(OR=8.24, [95% CI: 1.28 ~ 53.13], *p* = 0.027), and lower T2WI SI (OR=1.00, [95% CI: 0.99 ~ 1.00], *p* = 0.174) were identified as significant predictors of PCa. And T1 ≤1248.35ms (OR=8.79, [95% CI: 0.67 ~ 115.58], *p* = 0.098), higher SVR(OR=1.28, [95% CI: 1.06 ~ 1.54], *p* = 0.009), higher DWI SI(OR=1.04, [95% CI: 0.99 ~ 1.10], *p* = 0.120) and lower ADC (OR=0.00, [95% CI: 0.00 ~ 0.14], *p* = 0.011) for csPCa (**Table 3**). Nomograms were developed based on the final logistic regression models to assess the risk of PCa and csPCa (**Figures 4C, D**), achieving AUCs of 0.93 (95% CI: 0.86 ~ 1.00) and 0.96 (95% CI: 0.91 ~ 1.00), respectively. DCA demonstrated superior benefit across most threshold ranges. Calibration plots of predicted probabilities for PCa and csPCa showed excellent predictive accuracy (**Figure 5**).

**Table 3:** Lesion-level univariable and multivariable analyses for all prostate cancer and clinically significant prostate cancer in PZ.

Variable	Prostate Cancer		Variable	Clinically Significant Prostate Cancer	
	Multivariable Analysis			Multivariable Analysis	
	OR	*p* value		OR	*p* value
Category to Upgraded lesions			T1		
Yes	Reference		>1248.35ms	Reference	
No	3.33 (0.95 ~ 11.66)	**0.045**	≤1248.35ms	8.79 (0.67 ~ 115.58)	0.098
TIC			SVR	1.28 (1.06 ~ 1.54)	**0.009**
Type I and II	Reference		DWI signal intensity	1.04 (0.99 ~ 1.10)	0.120
Type III	4.82(0.78 ~ 29.64)	0.090	ADC	0.00 (0.00 ~ 0.14)	**0.011**
T1					
>1248.35ms	Reference				
≤1248.35ms	8.24(1.28~ 53.13)	**0.027**			
T2WI signal intensity	1.00(0.99~ 1.00)	0.174			

Data in parentheses are 95% CIs. OR, odds ratio; SVR, surface to volume ratio; PZ, peripheral zone. Values in bold indicate statistical significance (*p* < 0.05).

## Discussion

As far as we know, this is the first prospective study to demonstrate the performance of clinical data combined with PI-RADS v2.1 and mpMRI features for prostate cancer detection according to the different prostate zones in PI-RADS category upgraded 3 and 4 lesions respectively, which always puzzled urologists in determining the necessity of biopsies for patients with ambiguous bpMRI findings.

Our study reveals a significant difference between upgraded and non-upgraded PZ lesions in detecting PCa and csPCa. This suggests that the upgrading rule—where a PZ lesion with a DWI score of 3 (that dominant sequence for PZ) shows positive dynamic contrast enhancement, may not play a crucial role in determining PZ cancer rates. Our results are consistent with previous studies (14, 21), one of these studies reported the csPCa rate for upgraded PZ PI-RADS 4 lesions was lower than their non-upgraded counterparts (27% [37 of 136] vs 43% [58 of 136], *p* = .01), despite our smaller sample size (14).

However, PCa and csPCa rates were comparable between upgraded and non-upgraded TZ PI-RADS 3 and 4 lesions. This suggests that the upgrading rule based on prominent DWI signal features may improve the identification of PCa and csPCa in TZ lesions and improve the overall specificity of PI-RADS v2.1. A previous study reported a low prevalence of PCa in TZ PI-RADS 3 upgraded lesions (28% for any PCa and 8% for csPCa), which was favorable compared to csPCa detection rates in conventional TZ score 3 lesions (22). Although csPCa rates in PI-RADS 3 lesions were lower in our study, we validated the DWI upgrading rule introduced in PI-RADS v2.1 for atypical lesions, showing similarly significant PCa detection rates at TPUS biopsy compared to conventional T2-weighted TZ score 3 lesions.

Given that the lesions in different prostate zones exhibit significantly different detection rates for PCa and csPCa, we analyzed and developed various nomogram models using mpMRI and clinical data to predict PCa and csPCa based on prostate zonal anatomy. In predicting PCa, the highest diagnostic performance was observed in the PZ model, followed by the TZ model and the clinical combined with PI-RADS. In the PZ, PI-RADS non-upgraded lesions were more likely to be PCa-positive, reaffirming that the upgrading rule was unnecessary in our study. However, TIC type III(wash-out pattern) demonstrated excellent diagnostic performance for both PCa and csPCa. A previous study reported a contradictory finding that the mean early-phase DCE signal (derived from DCE) is predictive for PZ, but not for TZ (23). This discrepancy may be attributed to differences in DCE image analysis methods. The wash-out time-intensity curve has been shown to predict malignancies in the breast and pancreas (24, 25).

Recent studies (26, 27) have demonstrated quantitative parameters such as T1, T2 and PD values could be measured reproducibly, showing high specificity for PCa detection across different vendors, despite variations in repetition time and echo spacing. Although T1, T2, and PD values in different prostate zones, a general trend has been observed: higher-grade prostate cancers tend to have lower T1, T2, and PD values. In this study, we adopted cutoff values of T1, T2 and PD from a previous study (18),which used the same manufacture, MRI sequence, and postprocessing software. Notably, T1 relaxation time was significantly associated with PCa in both TZ and PZ. while PD could reliably differentiate csPCa in TZ. Our study demonstrates that synthetic MRI can also effectively differentiate PCa in PI-RADS category 3 and 4 lesions.

Although subjective analysis of T2WI features, such as lenticular shape, margin, and T2WI-rim, can diagnose TZ PCa with high specificity, it highly depends on the observer`s experience and exhibits only moderate inter-reader agreement. Shape metrics provide quantitative data regarding tumor shape and margins (28). Our study found that none of the quantitative shape parameters were able to distinguish PCa from benign prostate tissue. Similarly, another study failed to identify any specific lesion shape associated with csPCa (14). However, Krishna et al. demonstrated that quantitative shape features of circularity and convexity could accurately differentiate transition zone PCa from BPH lesions (28). Further studies exploring the association of more robust first-order texture features with cancer detection rates in lesions might be helpful (29).

This research has several limitations that should be noted. First, the number of participants in each subgroup was unbalanced. Second, since only targeted biopsies were considered, no significant association was observed between the prostate biopsy approach and csPCa detection rate when all biopsy indications were taken into account (30). Third, achieving precise histological–radiological correlation remains a significant challenge in prostate MRI research. The ROI region and the pathological area may not perfectly match, leading to unavoidable systematic errors despite different sampling approaches. However, in this study, we believe this type of mismatch was minimized by employing anatomical landmarks for prostatectomy specimens, a standardized scheme for TPUS-guided biopsy, and MRI-guided biopsy. Finally, a larger sample size and multicenter study are needed in the future studies.

## Conclusion

In this study, two key objectives were achieved. First, we demonstrated that the upgrading rule is applicable in the TZ but not in the PZ. Second, we developed nomograms based on mpMRI features to predict the probability of PCa and csPCa in different zones. These nomograms have the potential to assist in clinical decision-making. Future studies should aim to validate these models in larger cohorts and across external centers.

## Data Availability

The original contributions presented in the study are included in the article/**Supplementary Material**. Further inquiries can be directed to the corresponding author.
