# Supplementary material for: Prospective evaluation of mpMRI-derived nomograms for detecting prostate cancer in PI-RADS v2.1 upgraded and non-upgraded lesions
Source: Front Oncol. 2025 Jun 4;15:1510049. doi: 10.3389/fonc.2025.1510049 (PMC12173890; doi:10.3389/fonc.2025.1510049)
Supplement: Supplementary file 1 [file Table1.docx]

| **sTable1. Multiparametric MRI acquisition parameters** | | | | | | | | |
| --- | --- | --- | --- | --- | --- | --- | --- | --- |
| Sequence | TR(msec/  TE(msec) | FOV (mm) | Matrix | FA (degree) | Thickness  (mm) | b Value (s/mm^2^) | NEX | Time (min) |
|  |  |  |  |  |  |  |  |  |
| T_2_-weighted Imaging | 3000/105 | 200×200 | 384×384 | 111 | 3 | - | 2.5 | 3:25 |
| Diffusion Weighted Imaging | 4234/98.6 | 380×380 | 130×130 | 90 | 4 | 1000,2000,3000 | 3, 7, 14 | 5:36 |
| Dynamic Contrast Enhanced MRI | 5.9/5.1 | 340×340 | 300×300 | 15 | 1.1 | - | 0.71 | 3:42 |
| Synthetic MRI | 4000/14,90.7 | 260×208 | 320×256 | 90 | 4 | - | 2 | 7:44 |
| Note－TR=repetition time, TE=echo time, FOV=Field of View, FA=Flip Angle, NEX=number of excitation. | | | | | | | | |
